# Severe Heterotopic Ossification following Total Knee Replacement

**DOI:** 10.1155/2014/265489

**Published:** 2014-11-05

**Authors:** Alexander L. Dodds, Gregory C. R. Keene

**Affiliations:** Sportsmed SA, 32 Payneham Road, Stepney, Adelaide, SA 5069, Australia

## Abstract

Although the incidence of minor heterotopic ossification is probably higher than what is usually expected, severe heterotopic ossification (HO) is an extremely rare event following total knee replacement surgery. We present the case of a 66-year-old woman who initially had achieved an excellent range of motion following bilateral uncemented rotating platform total knee replacement, before presenting with pain and loss of range of motion at 2 months after surgery. Severe HO was diagnosed on X-rays. Treatment consisted of nonoperative measures only, including physiotherapy with hydrotherapy and anti-inflammatories. She eventually regained her range of motion when seen at 8 months after operation. This case illustrates that nonoperative treatment without the use of radiotherapy or surgery can be used to safely resolve stiffness caused by HO after total knee replacement.

## 1. Introduction

Severe HO following total knee replacement is a rarely reported event, although more minor HO is probably more common. In the small number of cases where severe HO of this nature has been reported in the orthopaedic literature, a variety of different treatment regimens have been suggested. These include conservative treatment in the form of physiotherapy and nonsteroid analgesics, manipulation under anaesthetic, use of radiotherapy, arthroscopic release of adhesions, and revision of the total knee replacement.

## 2. Case Presentation

**Figure 1:**
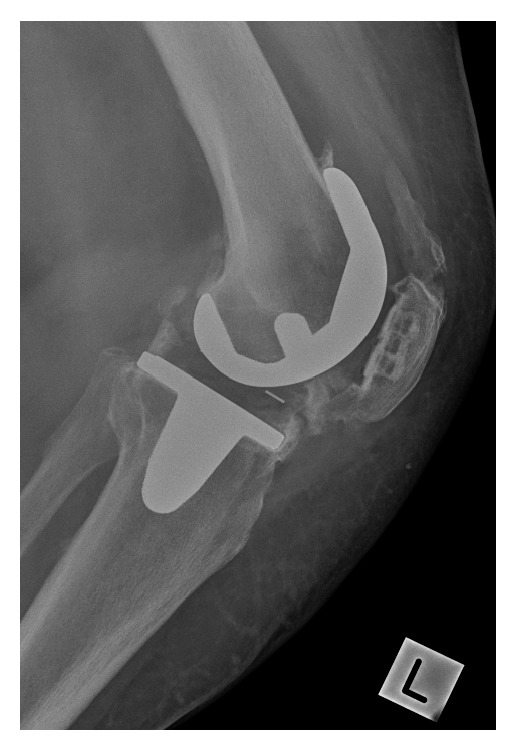
Postoperative radiographs of the total knee replacement at 8 months showing the severe heterotopic ossification.

**Figure 2:**
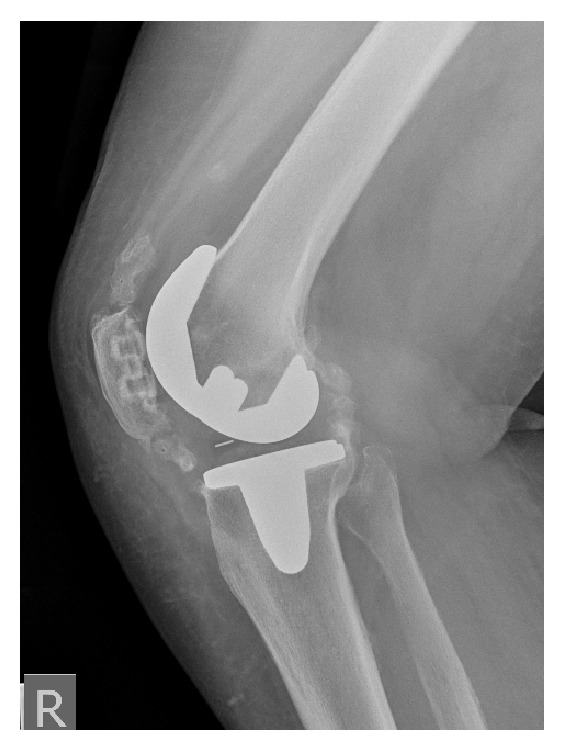
Postoperative radiographs of the total knee replacement at 8 months showing the severe heterotopic ossification.

**Figure 3:**
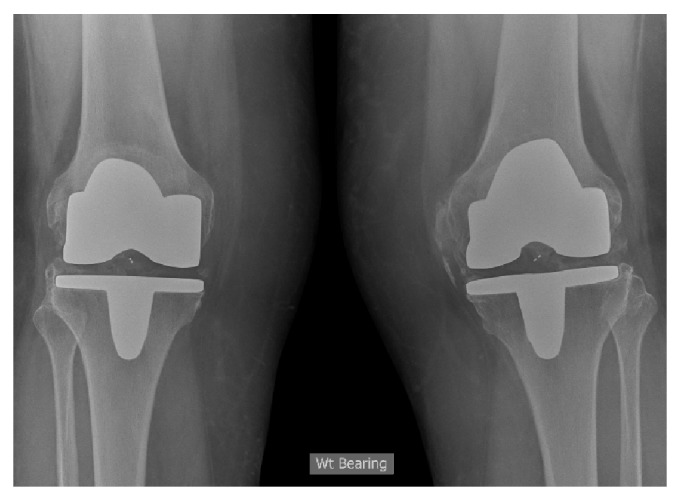
Postoperative radiographs of the total knee replacement at 8 months showing the severe heterotopic ossification.

A 66-year-old woman presented with severe osteoarthritis of both knees. Her weight was 89 kg and body mass index was 33. She had no other medical or surgical history. She subsequently underwent bilateral simultaneous total knee replacement surgery under general anaesthesia and with the use of tourniquet throughout. A standard medial parapatellar incision was used. A rotating platform prosthesis (LCS, Depuy, Warsaw) with uncemented femoral and tibial components was utilised, and the patellae were resurfaced with a cemented all polyethylene patella components. A satisfactory intraoperative result was obtained in both knees with a range of movement from 0 to 125. The deep layer of the medial collateral ligaments was partially released from the tibia for access during the procedure but no other soft tissue releases were required. The patient made an uneventful initial postoperative recovery and was discharged at day five after surgery. At 2 weeks after operation, she had obtained a range of motion of 0 to 90. At 10 weeks after operation she presented with a range of motion of 30 to 75 affecting the left knee only. By 12 weeks, she had decreased this range of motion of 40 to 75 with the left knee and 5 to 75 affecting the right knee. She had suffered no trauma to the knee. Blood tests revealed a CRP of 9, ESR of 85, and WCC of 4.7. Aspiration of the joint did not reveal any evidence of infection. X-rays showed calcification around the knee including the quadriceps tendon (Figures 1, 2, and 3). After a review of the literature, a decision was made to treat the loss of range of motion nonoperatively, with nonsteroidal anti-inflammatory medication (Diclofenac 50 mg tds) and other regular types of analgesia and physiotherapy. Physiotherapy consisted of weekly session focusing on range of movement exercises with additional sessions of hydrotherapy. Symptoms gradually improved and by 8 months she had regained a range of motion of 10 to 100 degrees in the left knee and 5 to 105 degrees in the right knee.

## 3. Discussion

Severe HO following total knee replacement is a rare event, with few reported cases in the literature. We have illustrated that when this complication does occur, satisfactory outcomes can be achieved with conservative treatment without the need for further manipulation under anaesthetic or arthroscopic release of adhesions, although this has been rarely reported previously. Previous authors have also advocated the use of radiotherapy in combination with physiotherapy and NSAIDS, although the outcome in our case was satisfactory without this [1]. There is a reported case where HO was so severe that resection of the collateral ligaments and reconstruction with a hinged implant have been needed [2].

The incidence of HO following total knee replacement surgery is probably higher than is commonly recognised, although, in most cases, it is only minor. In previously published work, 25 of 63 primary knee replacements (39%) were found to have postoperative HO, mainly in the region anterior to the distal femur [3]. This area was thought to be due to damage to the cortex or periosteum occurring during the anterior femoral osteotomy. Correlation was made between the degree of osteophyte formation preoperatively and the incidence of postoperative HO. The presence of minor HO was not found to affect the range of motion at one year, and, in 64% of knees, it reduced with time.

The largest case series consists of 500 patients undergoing cemented total knee replacement, which included 35 patients with bilateral total knee replacement [4]. The incidence overall was 15% of patients who had >1 cm of new bone formation. Five patients had HO of more than 5 cm, and these patients tended to be heavier males and all of them had a deformity of greater than 15 degrees. Earlier new bone formation was associated with larger amounts of new bone.

A retrospective review of 98 total knee replacements found an incidence of minor HO of 26% [5]. Six of the nine patients who developed HO had bilateral total knee replacements. Other risk factors for HO formation included elevated lumbar spinal bone mineral density and preexisting HO in other joints. The incidence of HO did not affect subjective outcome.

A case series of 221 cementless total knee replacements found a lower incidence of HO of 5% [6]. This may have been because all patients received high doses of aspirin after the operation as thromboprophylaxis. HO had developed by a mean period of 5 weeks, and size increased by a mean period of 9 weeks. The HO was only >5 cm in 1 knee. Risk factors for HO ossification were osteoarthrosis and the presence of a postoperative effusion. The presence of HO did not affect outcome.

## 4. Conclusion

Although the incidence of HO after total knee replacement is probably higher than what is commonly expected, with reported case series incidence of between 5 and 39% [3–6], the incidence of severe HO is much rarer with only a small number of reported cases. Risk factors for the development of HO in our case may have included increased body mass index and bilateral surgery. An acceptable result was achieved via nonoperative measures including anti-inflammatories and other types of analgesia and physiotherapy and hydrotherapy.
